# Evolution under intensive industrial breeding: skull size and shape comparison between historic and modern pig lineages

**DOI:** 10.1098/rsos.241039

**Published:** 2025-02-05

**Authors:** A. Haruda, A. Evin, F. Steinheimer, R. Schafberg

**Affiliations:** ^1^ Central Natural Science Collections, Martin Luther University Halle-Wittenberg, Halle (Saale), Germany; ^2^ Research Laboratory for Archaeology and the History of Art, University of Oxford, 1 South Parks Rd, Oxford OX1 3TG, UK; ^3^ ISEM, Univ Montpellier, CNRS, IRD, Montpellier, France

**Keywords:** *Sus scrofa*, geometric morphometrics, breeding, morphology, convergence

## Abstract

Domestication and subsequent human-induced selection has enhanced profound changes in animal morphology. On modern domestic pigs, those transformations encompass not only overall increases in body size but also modifications in skull morphology. While skull morphological differences between modern domestic pigs and wild boar are relatively well-documented, less understood is the variation and underlying mechanisms associated with intensive breeding. In this study, we investigated the rate and direction of phenotypic change of skull morphology using a unique dataset that includes two lineages of German domestic pig that were subjected to similar intensive industrial selection pressures throughout the twentieth century, alongside contemporaneous populations of German wild boar. Size and shape variation of 135 specimens was quantified through geometric morphometrics, with 82 three-dimensional landmarks. We find expected differences in skull shape between wild and domestic pigs, but also convergence between the two domestic lineages through the century of directed breeding, despite population segregation. Our results suggest that cranial morphologies have rapidly responded to selection pressure that is independent of genetic isolation. This also suggests that pig morphotypes quickly reflect human agency and impact upon domestic animal phenotypes, revealing a pathway to investigate early human breeding activity in ancient and historical contexts.

## 1. Introduction

Since the Neolithic revolution that began approximately 10 000 years ago, humans have been modifying and controlling a diversity of domestic animals, including pigs. With the advent of captivity, domestication and subsequent human-driven selection, the reduction of natural selection within a human-controlled context has impacted species phenotypes. Domestication experiments with wild foxes have shown that phenotypic variation can significantly increase via human selection within just a few generations [1,2]. Modern and ancient DNA studies of many domestic animals have revealed the emergence of variability in skull shape [3–5], coat colour [6,7] and other morphological characters such as the increase in vertebrae number in domestic pigs [8,9]. However, the rate at which phenotypes would have responded to human control remains unclear [10].

Indeed, while domestic animals often display clear phenotypic differences from their wild ancestors, existence of admixture through cross-breeding between domestic and wild animals, as well as between different domestic populations, complicates our understanding of human modification of these taxa [11–13]. Historical records from Europe indicate that the domestic animal phenotypic diversity in this region dramatically increased during the periods of the Agricultural and Industrial Revolutions of the late eighteenth and early nineteenth centuries. This increase was at least partially driven, especially in pigs, by the incorporation of novel breeds, such as those from China, in order to increase animal productivity and to conform to cultural ideas of the ‘ideal’ animal that fit with economic and industrial practices [14–18].

During this period gentleman farmers, such as Robert Bakewell (1725−1795), began to improve local sheep and cattle through selective breeding. Pigs, too, were subject to intense selective breeding throughout the eighteenth century [17,19–21]. Swine imported from southern China were crossed with many European breeds, such as the Berkshire, in pursuit of precocity (early maturation), larger carcass size and large litters [22–24]. However, some local breeds, such as the now extinct ‘*Deutsches Weideschwein*’, were maintained without Asian influence and continued to exhibit a phenotype similar to local wild boar. The never-ending pursuit of increasing profitability throughout the nineteenth and twentieth centuries in regional and national European markets challenged traditional subsistence practices, encouraging the spread of hybridization, cross-breeding experiments and specialization of farming techniques [25]. The focus extended beyond controlling breeding to also include animal nutrition, as pigs transitioned from largely free-range recyclers of human refuse to consumers of highly processed grain mixed with oil-seed crops [25,26]. This approach became a focus of national administrative attention at the turn of the twentieth century as many countries integrated into global markets [27,28]. Today, modern industrial livestock supply chains are highly interdependent and segmented. Isolated mother and father lines of specific pig lineages are maintained to create F1 and F2 hybrid flocks.

**Figure 1.**
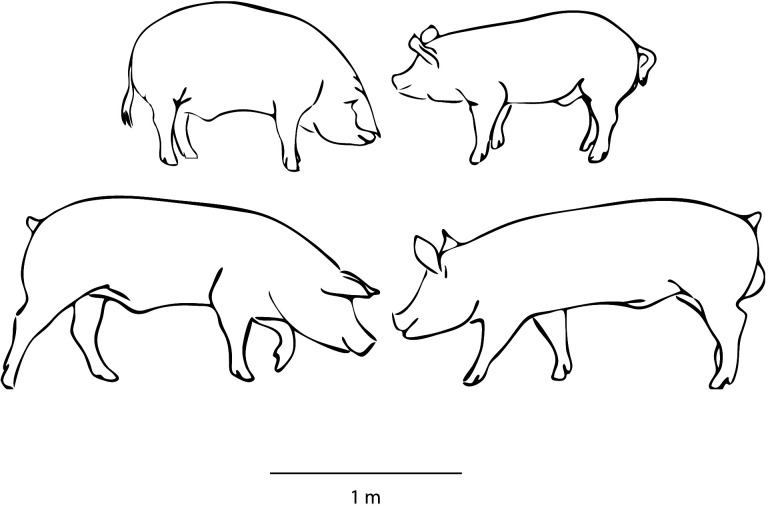
Silhouettes of adult males of two domestic pig lineages at approximately 1920 (top), and from 2015 (bottom). Deutsches veredeltes Landschwein/Deutsche Landrasse (DL, left), Deutsches Edelschwein (DE, right). Note the difference between ear orientation, tails (modern tails are docked) and conformation. Sketch by R. Schafberg.

In Germany, there are still two main lineages of domesticated pigs in commercial pork production today: the *Deutsche Landrasse* (formerly known as *Deutsches Landschwein* or *Deutsches veredeltes Landschwein*, here abbreviated as DL), and the *Deutsches Edelschwein* (known as the ‘noble’ or German large white pig, here abbreviated as DE) [29]. The DL originated from a local type of pig from northwest and central Germany, the *Marschschwein*. Similarly, the DE also descends from the *Marschschwein* but was improved by cross-breeding with English Yorkshire and Middle White lineages. These improvements were first documented in the second half of the nineteenth century, and received German state support for breeding purposes in special research centres at the beginning of the twentieth century, such as in Halle (Saale) [27]. At that time, the DL and the DE were distinct, marked by a difference in body conformation, ear, tail and skull shape (figure 1). Throughout the twentieth century, these breeding lines were continuously and separately maintained. At the end of the 1950s, breeding focus shifted towards meat (muscle) production rather than total fat content of the carcass. This significant transformation led to the ‘*Deutsche veredeltes Landschwein*’ renamed as *Deutsche Landrasse*. While the DE also experienced improvements to move towards the same production goal, the impact was relatively less pronounced and the lineage retained its original name [15,16,18,29].

Through the twentieth century, livestock breeding techniques have become more efficient to meet the demands of the market, with a particular focus upon developing phenotypic traits such as carcass size and a reduced proportion of fat relative to muscle, but not upon traits such as colour, head shape and tail and ear shape [30,31]. This unique industrial context provides an opportunity to investigate the rate of phenotypic change under intensive selective pressure, creating a case study for exploring the rate and amplitude of phenotypic response to microevolutionary processes.

This study investigates changes in the size and shape of pig skulls from both wild and domestic German pig populations dating from the turn of the twentieth century (historic) and the twenty-first century (modern), thus roughly spanning 100 generations of pig breeding. Two domestic pig lineages were studied: the *Deutsches Edelschwein* (DE) and the *Deutsche Landrasse* (DL), along with a control group of wild boar. The skull size and shape were quantified through a three-dimensional landmark-based geometric morphometric approach. The aim is to determine whether selection for industrial production has resulted in similar skull characteristics, i.e. convergence, in both domestic populations through time and to what extent these traits have covaried.

## 2. Material and methods

### 2.1. Data collection

A total of 135 skulls were studied including both historic and modern specimens from wild boar, Deutsche Edelschwein (DE), and Deutsche Landrasse (DL) lineages from Germany (table 1, electronic supplementary material, SI). Historic domestic specimens originate from livestock rearing experiments that took place before the Second World War and were focused upon identifying and improving traditional German pig landraces and are curated at the Domestic Animal Collection, Martin Luther University Halle-Wittenberg, Germany. Historical wild boar skulls originate from material collected at the beginning of the twentieth century prior to the Second World War across northeast Germany and modern-day Poland (Prussia) and are curated at the Museum für Naturkunde, Berlin. All modern wild and domestic specimens were collected from Mecklenburg Western Pomerania in Germany between 2017 and 2019. Domestic pigs were raised by breeders that specialize in raising pedigree pigs for commercial purposes. Modern specimens were measured, slaughtered and processed according to standardized procedures at the Research Institute for Farm Animal Biology in Dummerstorf, Germany, and the skeletal remains were processed at the Museum für Naturkunde, Berlin. All modern pigs were slaughtered after at least 2 years of age. The historical skulls, all from adults, were selected based upon tooth eruption to account for age-related osteological variation. All studied historical specimens had at least a partial eruption of the third mandibular molar, which erupts between 18 and 24 months of age, although root formation in wild boar continues beyond this point [32–34].

**Table 1.** Skull specimens (*N *= 135) included in the study from two collections, the Domestic Animal Collection, Martin Luther University Halle-Wittenberg as well as the collection of the Museum für Naturkunde, Berlin. DE = Deutsches Edelschwein, DL = Deutsche Landrasse.

lineage	historic	modern	total
DE	28	20	48
DL	23	16	39
wild boar	33	15	48

Skulls were three-dimensionally scanned using an Artec Eva structured light scanner. The surface scan data were processed and three-dimensional models assembled using Artec Studio 15 (Artec 3D, Luxembourg). Models were saved as PLY files without surface texture, and coordinates of 82 three-dimensional landmarks on the ventral and lateral aspects were acquired using a modified landmarking protocol based on Owen *et al.* [5] (see electronic supplementary material, SI: Landmarks, SI: Specimens) in IDAV Landmark software [35].

### 2.2. Geometric morphometrics and statistics

Landmark coordinates were superimposed using a generalized Procrustes analysis (GPA), which is a procedure that centres, scales and rotates landmark configurations [36]. This was done considering object symmetry using paired landmarks in the package Morpho 2.11 for R [37,38]. To explore shape variation across the entire dataset, a principal component analysis (PCA) [39,40] was performed on the Procrustes residuals using the package geomorph 4.0.6 in R [41]. Visualization of the shape changes associated with the principal component axes and average shape for each group of interest was performed using the warpRefMesh function in geomorph and meshDist function in Morpho.

In order to understand and describe any convergence of phenotype through time, analysis focused on characterizing and describing variation within each lineage across the two time periods in reference to the entire dataset including wild boar. This was then run again on a dataset that included only domestic lineages in order to more closely examine the variation present within this dataset. Sexual dimorphism was not investigated as the sample sizes were too small to be suitable for statistical testing.

**Figure 2.**
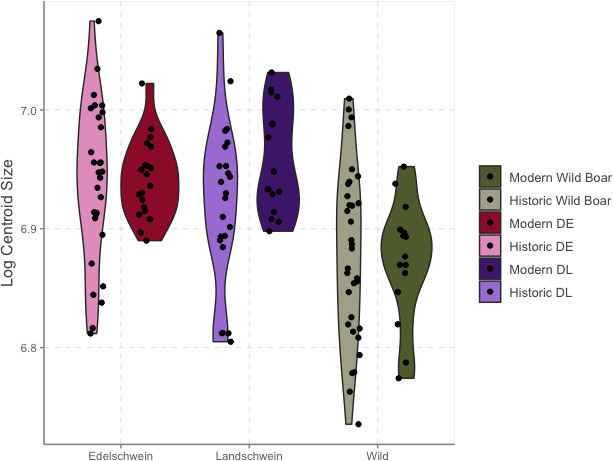
Violin plot of log centroid size of wild and domestic pigs, separated by historical time period. DE = Deutsches Edelschwein, DL = Deutsche Landrasse.

Size differences within each lineage between historic and modern groups were explored using log centroid size (CS) which was visualized by boxplots (figure 2). A nested approach using a two-way ANOVA with a permutation procedure (999 iterations) using the procD.lm function in geomorph 4.0.7 tested the homogeneity of variation through time among lineages [42]. Pairwise statistics were generated using RRPP 1.4 from this model to explore differences between means and variances (table 2) [42].

**Table 2.** Results of analysis of centroid size using pairwise comparisons following two-way ANOVA for populations from each time period within each lineage for the entire dataset, including wild boar. Distance values from the pairwise statistics are Euclidean distances between least-square means, effect sizes (*Z*) are derived from the *F* distributions, and *p* is for *p*-value. DE = Deutsches Edelschwein, DL = Deutsche Landrasse.

pairwise comparision between means	distance	effect size (*Z*)	*p*
historic DE: modern DE	0.0046	−1.0084	0.831
historic DL: modern DL	0.0362	1.2939	0.112
historic wild boar: modern wild boar	0.0058	−0.8548	0.797
**pairwise comparison between variances**			
historic DE: modern DE	0.0032	1.9547	0.015
historic DL: modern DL	0.0025	1.2461	0.1
historic wild boar: modern wild boar	0.0027	1.4448	0.067

group variances	variance		variance
historic DE	0.0042	modern DE	0.0010
historic DL	0.0044	modern DL	0.0019
historic wild boar	0.0050	modern wild boar	0.0024

Shape differences between historic and modern populations were investigated by analyses of variance on shape data (i.e. Procrustes coordinates) using procD.lm (geomorph) and pairwise (RRPP) functions using a 95% confidence interval (table 3) [42]. This analysis was run on both the entire dataset including wild boar (A) and a reduced dataset containing only domestic data (B), in order to more closely examine the variation contained in a dataset without the impact of including wild boar as a control.

**Table 3.** Results of analysis of shape (Procrustes coordinates) using a two-way ANOVA and pairwise comparisons for populations from each time period within each lineage for the entire dataset, including wild boar. Dataset A includes wild boar as a control, while Dataset B contains only domestic pigs. Degrees of freedom as abbreviated as (d.f.), sum of squares (SS), mean squares (MS) and *R*^2^ represents the percentage of variation in a given model. Distance values from the pairwise statistics are Euclidean distances between least-square means, effect sizes (*Z*) are derived from the *F* distributions and *p* is for *p*-value. DE = Deutsches Edelschwein, DL = Deutsche Landrasse.

Dataset A: domestic pigs and wild boar							
**two-way ANOVA with Type II SS**	d.f.	SS	MS	*R* ^2^	*F*	effect size (*Z*)	*p*
lineage	2	1.320	0.660	0.68569	154.212	5.607	0.001
time period	1	0.022	0.022	0.01145	5.149	4.035	0.001
time period: lineage	2	0.025	0.012	0.01278	2.877	3.738	0.001
residuals	129	0.552	0.004	0.28679			

**pairwise comparison between means**	distance	effect size (*Z*)	*p*	**pairwise comparison between variances**	distance	effect size (*Z*)	*p*
historic DE: modern DE	0.0480	3.2585	0.002	historic DE: modern DE	0.0020	1.9981	0.017
historic DL: modern DL	0.0422	1.8934	0.025	historic DL: modern DL	0.0013	1.0989	0.136
historic wild boar: modern wild boar	0.0179	−4.5454	1	historic wild boar: modern wild boar	0.0006	0.1653	0.458

**group variances**		variance				variance	
historic DE		0.0067		modern DE		0.0047	
historic DL		0.0047		modern DL		0.0034	
historic wild boar		0.0024		modern wild boar		0.0018	


Dataset B: domestic pigs							
**two-way ANOVA with Type II SS**	d.f.	SS	MS	*R* ^2^	*F*	effect size (*Z*)	*p*
lineage	1	0.104	0.176	19.458		3.924	0.001
time period	1	0.029	0.050	5.530		3.574	0.001
time period: lineage	1	0.013	0.023	2.522		2.411	0.005
residuals	83	0.442	0.005	0.751			

**pairwise comparison between means**	distance	effect size (*Z*)	*p*	**pairwise comparison between variances**	distance	effect size (*Z*)	*p*
historic DE: modern DE	0.0478	1.1440	0.1230	historic DE: modern DE	0.0020	2.0626	0.012
historic DL: modern DL	0.0419	−0.4960	0.6880	historic DL: modern DL	0.0013	1.1649	0.138

**group variances**		variance				variance	
historic DE		0.0066		historic DL		0.0047	
modern DE		0.0046		modern DL		0.0034	

In the context of this study, convergence will be recognized if the two modern populations of the domestic lineages fall closer to each other than to their historic relatives in the multivariate shape space [43]. Shape convergence was explored using a multidimensional convergence index (MCI) [43,44]. This index was calculated as the ratio of the Procrustes variance within the modern populations and within their ancestral, historic populations. The obtained MCI values were compared with a random distribution obtained from 999 MCI values based on randomized historic/modern attributions. An MCI value above 1 will indicate convergence.

## 3. Results

### 3.1. Centroid size

Wild boar skulls are consistently smaller than domestic pigs for both time periods (figure 2). The variation within lineage appeared more important than the variation between periods, with no significant interaction between the two factors (two-way ANOVA: Time Period: *R*^2^ = 0.013, *p* = 0.142; Lineage: *R*^2^ = 0.186, *p* = 0.001; Lineage by Time Period: *R*^2^ = 0.154, *p* = 0.29). Pairwise comparisons within each lineage found no significant difference in either means or variance through time, with the exception of the differences between size variance of the DE lineage (historic population variance = 0.0042, modern population = 0.0019, *p* = 0.015) which indicated a reduction in variance in this population through time that was not seen in the DL or wild boar populations (table 2). As size was not a significant source of variation within lineages, it was retained for downstream analyses of shape.

### 3.2. Shape

**Figure 3.**
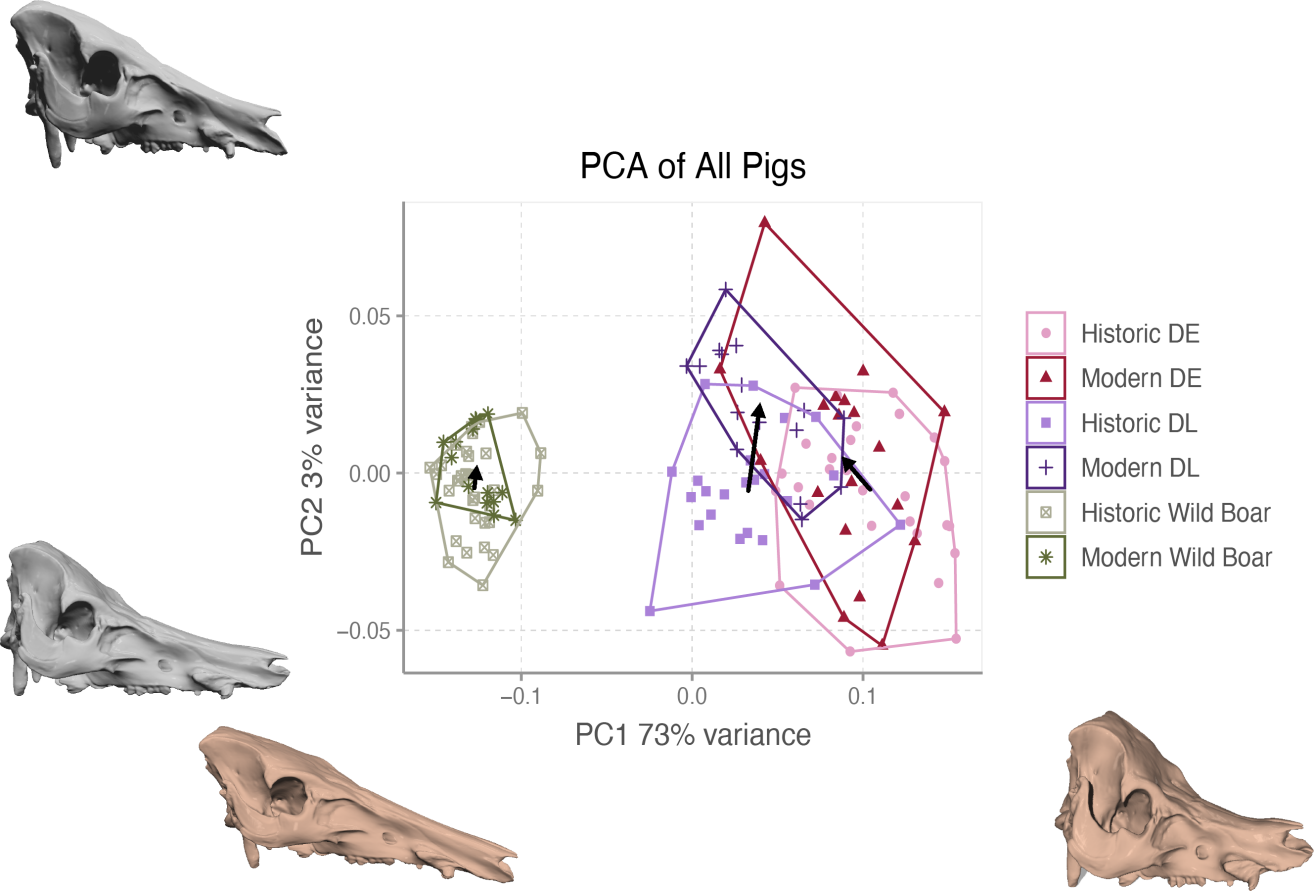
Principal component analysis of skull shape variation for the entire dataset. Arrows link population means from each time period within lineages. Warped skulls were created from an average skull shape to visualize morphology from the end of each principal component axis. Variation encompassed by the first principal component is displayed as sepia coloured models while grey models reflect variation from the second principal component. DE = Deutsches Edelschwein, DL = Deutsche Landrasse.

A principal component analysis of the entire dataset revealed a clear separation between wild and domestic pigs independently of the period along the first axis of the principal component analysis (PC1 73% of total variance) (figure 3). Domestic pig skulls possess shorter facial portions in comparison with cranial portions and are overall more compressed along the anterior/posterior axis. Variation within domestic pigs in this dataset is largely contained within the second axis (3% variation), with historic and modern populations varying along this axis. Modern domestic pigs have positive values on the second axis and have lost some of the characteristic concavity in the frontal bones that was associated with the historic domestic skull phenotypes, which is evident in the grey warped reference skulls that depict variation along this principal component (figure 3).

Analysis of shape data shows that there is a significant effect of both breed and time period (table 3, Dataset A). The three lineages differ in their skull shape (*F* = 154.212, *Z* = 25.607, *p* = 0.001), and the wild boar populations are at least two times less diverse than populations from domestic lineages. Historic and modern wild boar do not differ in shape or variance. Conversely, there is significant difference between means of populations from different time periods within lineages of domestic pigs, and a significant reduction in shape variance for the DE lineage which parallels significant reduction in size variance (table 2). This shape change is evident in figure 3, in which arrows connecting means of each time period population within lineages show a change in shape for both DL and DE lineages, with the DE population displaying a change not just along PC2 but also along PC1, underlining a change in skull shape.

**Figure 4.**
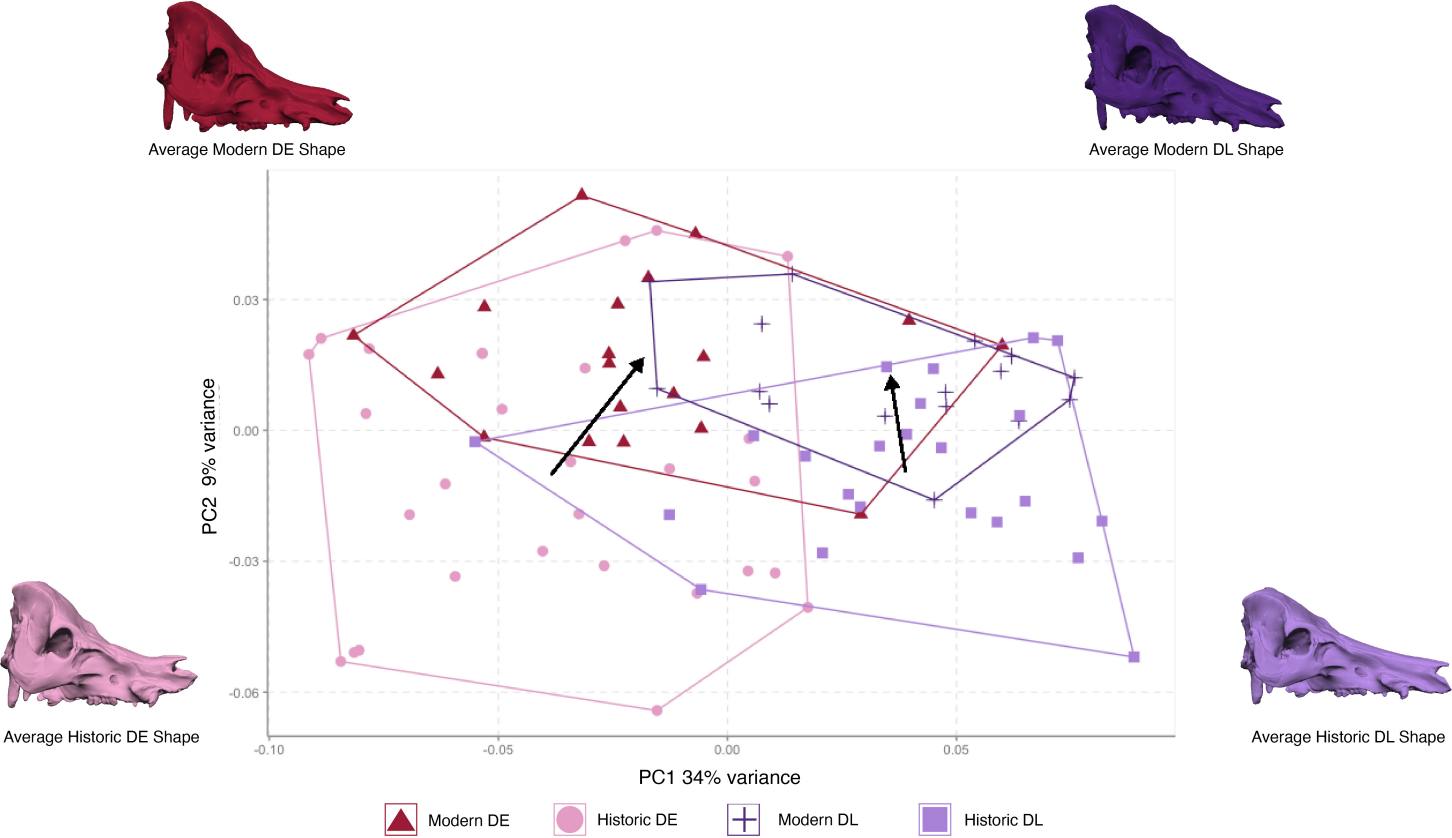
Principal component analysis of skull shape variation for only domestic lineages. Arrows link population means from each time period within lineages. Warped skulls were created from an average skull shape to visualize average morphology for each population. DE = Deutsches Edelschwein, DL = Deutsche Landrasse.

This change in shape within domestic lineages is explored in more detail in Dataset B, which removes wild boar. The resulting PCA details the change in shape through time for each lineage (figure 4). Lineages are separated along the first principal component (34% variation) while populations from each time period lie along the second principal component (9% variation). Skull models warped to the average shape for each population with no magnification show the subtle shape changes that occur within each lineage through time. The unique historic skull phenotypes clearly define each lineage, with the DE having shorter and more compressed nasal bones in comparison with the DL. Historic populations of both DE and DL pigs have a small upraised end of the nasal bone on the anterior end, which would result in a characteristic flat and upright nose on a fully fleshed animal. The nasal bone with an upright anterior end has disappeared in modern populations of both lineages. In addition, both lineages have lost the associated concavity in the frontal bone which was associated with this anterior nasal orientation. Morphological changes through time are more apparent in the DE lineage, where the loss of this feature is more pronounced. In addition, there is a change in orientation of the dorsal aspect of the skull through time for both lineages, with a flatter dorsal aspect of the cranial vault in modern populations, and a reduction in the degree of dorsal overhang of the nuchal crest.

**Figure 5.**
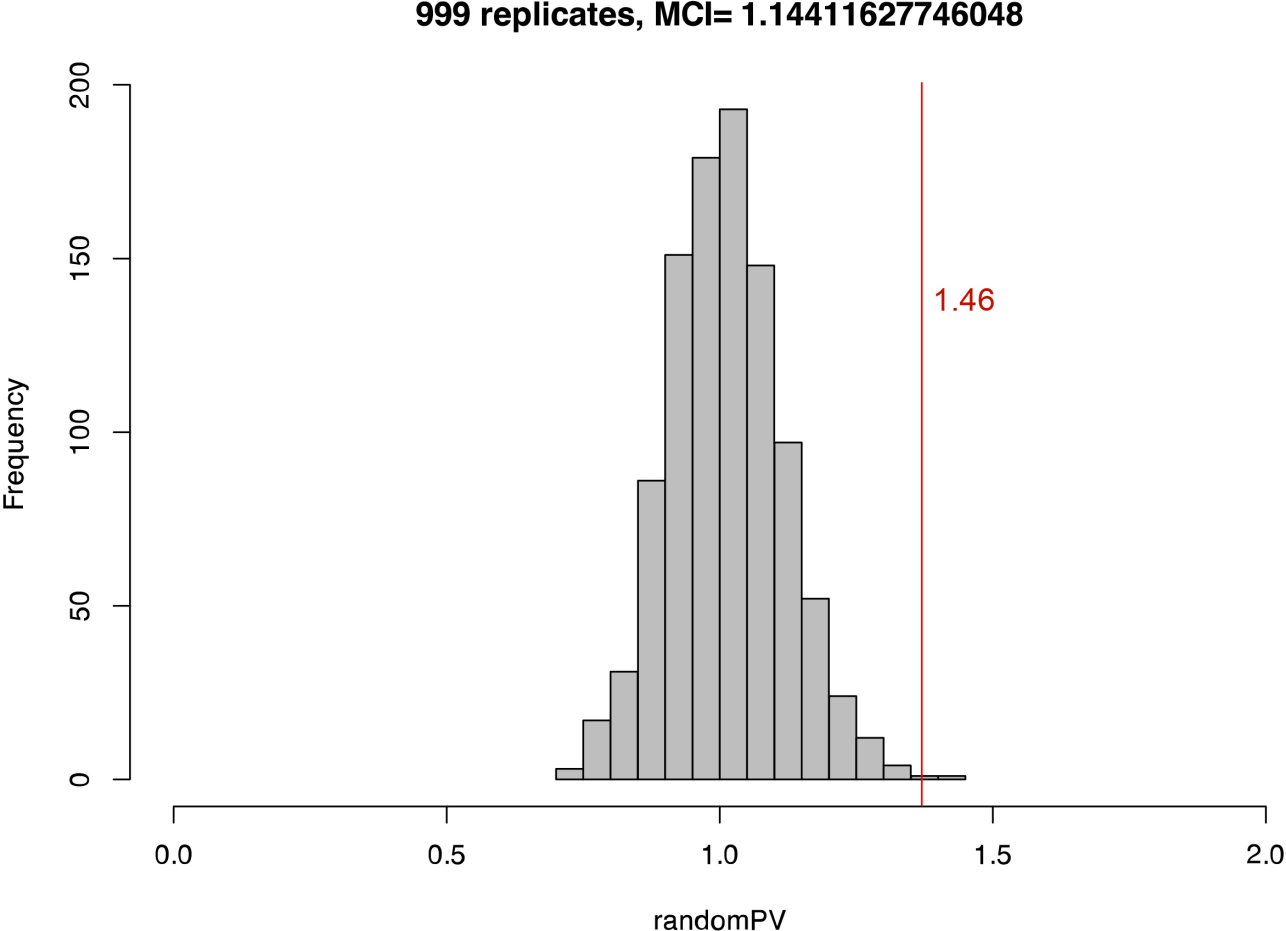
Multivariate convergence index (MCI) value shown as a red line plotted against a random set of 999 MCI values with an average value of 1.144 during whose computation the group attribution as modern or historic is randomized.

The four groups differ in their skull shape (interaction term Time Period: Lineage = *F *= 2.5522, *Z* = 2.411, *p* = 0.005), and for both lineages, the historic population was more diverse than the modern domestic populations. The PCA indicates that there appears to be a movement through time towards a similar skull morphology, with a loss of unique osteological morphological characters. A test for convergence on the domestic pigs resulted in an MCI value of 1.46 (*p* = 0.002), indicative of morphological convergence through time (figure 5).

## 4. Discussion

The results presented here reaffirm previous work on the morphological differences between wild boar and domestic pigs [5,45]. Temporally, there is little shift in mean size through time for all the studied lineages, including the wild boar. However, we can see that there is a loss of size variation for the DE through time and there is visible shape change as seen in figure 4. A region of genetic differentiation (SSC1) is syntenic to a region associated with cranial dimensions in dog breeds and was observed in modern European pigs in comparison with wild European boar and thus may have an influence on the variation in cranial size [8,46–49].

Historical evidence confirmed that selection in domestic pig populations during the period following the Second World War changed to quantitative productive traits such as sexual maturity, fertility and carcass size as well as lean muscle production [15,29,50]. Increasing meat consumption per capita and a drive to lower unit cost and increase profitability across the industrial livestock across Germany in the twentieth century drove increased rates of weight gain in animal populations [18,51]. This has resulted in individual animals reaching marketable weight (100 kg) at five months of age with daily weight gains averaging 800−900 g d^−1^ whereas between 1932 and 1935 market weight was not reached until 11 months of age with a daily weight gain of about 500 g d^−1^ [52,53].

These changes were accompanied by an increase in the overall length of individual pigs at slaughter, despite a reduction in slaughter age. Indeed, breeding for lean growth has been shown to result in an increase in withers height, and body and skull length, as well as smaller chest circumference and shoulder width [54]. The modern pigs in our study were measured after slaughter and were on average 161.0 cm (DL) and 156.6 cm (DE) in total length, while historic populations from the turn of the last century were 105.6 cm (DL) and 116.6 cm (DE). This increase in body length is mirrored in overall skull length with an increase from 26.8 to 40.7 cm (DL) and 25.2 to 39.3 cm (DE) [55,56], yet our results (table 2) show that there is no significant difference in centroid size of the skulls through time for both domestic populations, and thus centroid size is not a good proxy for estimating changes in overall animal size through time.

Of note are the differences in the skull centroid size of the wild boar in comparison with domestic pigs, which in our study shows that wild specimens are smaller. This follows the findings of other studies [45,57] although a single study found that wild European boars were larger than domestic pigs 100 years ago [5]. Our historic sample of wild boars comes from a geographical range that includes Mecklenburg Western Pomerania, where our modern wild boar was collected, but also includes areas of Poland, which were part of Prussia when collected in the late nineteenth and early twentieth centuries. Neaux *et al*. describe a smaller centroid size in wild-caught than in captive wild boars, with the latter resembling domestic breeds in their skull centroid size and shape [45]. The authors hypothesize that this is related to changes in masticatory forces that are associated with a reduction in foraging and rooting behaviour. Additionally, Albarella *et al.* classified their sample of Prussian wild boar skulls from the Museum für Naturkunde in Berlin as small in comparision with other Central European specimens [57] which was also noted by Groves [58]. We find our results and those of the above studies vary from Owen *et al.* [5], which is probably due to differences in sample selection criteria of Prussian wild boar from the collections of the Museum für Naturkunde.

Skull shape significantly shifts over a 100-year period with a change in shape of the frontal and nasal bones which relates to the degree of concavity or ‘dishing’ in the forehead, as well as a change in the orientation of the rear of the skull. The final morphological dished shape characteristic of many domestic lineages is not a paedomorphic trait, but instead a highly mutable phenotypic trait specific to domestic taxa, and therefore variation between lineages is not unusual [46,59,60]. However, this shape change is unexpected diachronically within these lineages given that there has been no directed selection pressure on skull morphology through time, and selection pressure upon both domestic lineages has been identical [5].

Changes in the orientation of the dorsal aspect of the skull have been noted in historic literature and are seen with breeding for sexual maturity and growth. The width of the head increased after crossing with Asian pigs, and the shortening of the skulls, specifically the frontal and occipital bones, seems to be associated with breeding for fattening ability and feed conversion [61]. Starvation–fattening experiments in pigs had already shown that feeding influences the growth of the skull and the expression of the shape, with young animals fed ad libitum displaying shorter skulls and dished profiles in comparison with littermates which had longer skulls and lacked a dished profile [62,63]. However, it is unclear if the change seen in our specimens may be related to changes to feeding regimes over our study period. Currently, many domestic swine are fed pellets from hoppers or troughs low to the ground, while at the turn of the last century, it was likely that there were more opportunities to find food by rooting or digging—yet the historic individuals were raised in a paved courtyard with no opportunity to engage in these behaviours. Thus we are unable to explain this variation in morphology due solely to changes in feeding regime or housing.

Both domestic and wild pig populations have been subject to increases in population size and intense human management. Wild boar populations significantly increased following a growth in habitat and availability in food sources, despite increasing pressure on the population by state-sanctioned culls [25]. These effects did not cause detectable morphological changes, even if this could be expected [45]. Parallel to this, domestic pig lineages were heavily bred in purpose-built industrial farms [25]. As pressure was applied for similar geno- and pheno-types that reflected post-war productive traits, other morphological features also converged over this relatively short time period. Our results suggest that a focus upon production traits rather than general conformation in modern lineages has interfered with maintenance of traditional breed types. However, the allometric characterization of ontogeny through time for each lineage is still poorly described in geometric morphometric terms. Multivariate analysis of linear measures indicates that there is no change in growth trajectory between wild and domestic pigs, and that isometry is more prevalent within the skull of domestic pigs than in wild boar, by contrast to heterochronic patterns of the growth trajectory and allometry in some domestic taxa, such as dogs. Furthermore, the frontal bone of domestic mammals is more variable than other cranial elements, although this is not related to a universal ‘domestication syndrome’, but rather to a lack of integration and increasing modularity [60,64].

Thus, the pressure from human selection over 100 generations is enough to significantly vary phenotypes in domestic pigs in comparison with contemporary local wild boar populations. This suggests a very strong response of phenotypes of these domestic animals to intensive selection and that lineage phenotypes are not fixed, despite isolation from other lineages. This is in concordance with the parallel proliferation of similar genes for fast growth in commercial pig populations alongside a harmful missense mutation that results in physiological problems in modern herds, such as a tendency to malignant hyperthermia [9,65]. Furthermore, a similar selection pressure on isolated populations of varying phenotypes results, at least for the pig lineages studied here, in a convergence of these phenotypes and loss of diversity between and within these breeds. A similar result is observed on the St Bernard’s and other dog breeds that show rapid morphometric changes over the last century [4,10], as well as within genetic studies of livestock populations [66].

A loss of phenotypic diversity between these two studied domestic lineages and convergence towards a similar morphotype is clear from our results, and parallels literature that explores a similar loss of genetic diversity over the past century [66,67]. While both breeds have been segregated for decades, they have been subject to identical breeding goals that have changed over this period to reorient from physical phenotype to physiological characteristics. This convergence towards a similar skull phenotype appears to reflect a reorientation from separate to unified breeding direction for these isolated lineages. This change in osteological morphology over a relatively short time period, historically and archaeologically speaking, is especially significant in relation to the lack of morphological change observed in wild boar populations across the same period. For archaeologists and historians, this work reveals a promising avenue to detect the impact of human breeding practices upon the domestic pig, as we have shown that the skull reflects changes in breeding goals, regardless of whether those goals were specifically directed at phenotype or instead at other, more difficult to detect characteristics, such as taste, profit and industrial efficiency.

## Data Availability

Original three-dimensional scans of this material are available at: https://opendata.uni-halle.de/handle/1981185920/113843. Original three-dimensional landmarks that compose this dataset are available at [68]. Supplementary material is available online [69].
